# Is Very High Thyroid Stimulating Hormone Level Required in Differentiated Thyroid Cancer for Ablation Success?

**DOI:** 10.4274/mirt.88598

**Published:** 2016-06-06

**Authors:** Zekiye Hasbek, Bülent Turgut

**Affiliations:** 1 Cumhuriyet University Faculty of Medicine, Department of Nuclear Medicine, Sivas, Turkey

**Keywords:** thyroid cancer, thyroid stimulating hormone level, radioiodine therapy

## Abstract

**Objective:**

Remnant ablation with radioactive iodine (I-131) is a successful form of treatment that aims to destroy the remaining residual tissue and/or metastatic tissue after total thyroidectomy in differentiated thyroid cancer (DTC) patients. High level of thyroid stimulating hormone (TSH) (≥30 mIU/L) is recommended for success of ablation treatment. In this retrospective study, our aim was to investigate whether the TSH levels at the time of ablation effect the success of radioactive iodine remnant ablation.

**Methods:**

Patients who were diagnosed with DTC, treated with bilateral total/near total thyroidectomy and who were referred for I-131 remnant ablation were included in this study. Patients with undetectable TSH-stimulated serum thyroglobulin (Tg) level, normal physical examination, negative results on whole body scan with I-131, and no evidence of neck lymph node metastasis on ultrasound were defined as disease-free. The correlation between TSH level at the time of ablation and ablation success was assessed.

**Results:**

Two hundred sixty one consecutive patients were included in the present study. Mean TSH level was 19.47±6 mIU/L in the 34 patients with TSH <30 mIU/L, while mean TSH level was 73.65±27 mIU/L in the 227 patients with TSH ≥30 mIU/L during I-131 remnant ablation. Ablation was unsuccessful in only one patient with TSH <30 mIU/L who had lung metastasis. Ablation was unsuccessful in 5.1% of patients with TSH ≥30 mIU/L. The effect of TSH level was not significant on ablation success (p=0.472).

**Conclusion:**

In conclusion, we think that a high TSH serum level alone is not a factor for the success of ablation. Age, presence of metastasis, extent of residual thyroid mass should also be considered. Especially, in the presence of metastatic tissue, obtaining adequate increase in TSH level is not always possible. The success of ablation at lower levels of TSH elevations may be sufficient for patients, and long-term hypothyroidism may not be required.

## Abstract (tr)

**Amaç:**

Diferansiye tiroid kanser (DTK), hastalarında total tiroidektomi sonrası radyoaktif iyot (I-131) ile remnant ablasyon kalan rezidü doku ve/veya metastatik dokunun yok edilmesi amacıyla kullanılan başarılı bir tedavi yöntemidir. Başarılı ablasyon tedavisi için yüksek tiroid simüle edici hormon (TSH) (≥30 mIU/L) seviyesi önerilmektedir. Bu retrospektif çalışmada ablasyon sırasındaki TSH düzeyinin radyoaktif iyot remnant ablasyonun başarısı üzerine etkisinin araştırılması amaçlandı.

**Yöntem:**

Bu çalışmaya DTK tanısı olan, bilateral total/totale yakın tiroidektomi ile tedavi edilen ve I-131 ile remnant ablasyon için yönlendirilmiş hastalar dahil edildi. Detekte edilemeyen stimüle-TSH serum tiroglobulin düzeyi, normal fizik muayene, I-131 ile tüm vücut tarama sonuçları negatif ve ultrasonda boyun lenf nodu metastazı bulgusu olmayan hastalar hastalıksız olarak kabul edildi. Ablasyon başarısı ile ablasyon sırasındaki TSH düzeyi arasındaki ilişki değerlendirildi.

**Bulgular:**

Mevcut çalışmaya ardışık 261 hasta dahil edildi. I-131 ile remnant ablasyon sırasında TSH düzeyi <30 mIU/L olan 34 hastada ortalama TSH düzeyi 19,47±6 mIU/L, TSH düzeyi ≥30 mIU/L olan 227 hastada ortalama TSH düzeyi 73,65±27 mIU/L idi. TSH <30 mIU/L olan bu hastaların yalnızca birinde ablasyon başarısızdı ve bu hastada akciğer metastazı vardı. TSH düzeyi ≥30 mIU/L olan hastaların ise %5,1’inde ablasyon başarısızdı. TSH düzeyinin ablasyon başarısına etkisi anlamlı değildi (p=0,472).

**Sonuç:**

Sonuç olarak, biz ablasyon başarısı için yalnızca yüksek serum TSH seviyesinin yeterli bir faktör olmayacağını düşünüyoruz. Yaş, metastaz varlığı, rezidü tiroid dokusunun büyüklüğü de dikkate alınması gereken faktörlerdir. Özellikle metastatik doku varlığında TSH seviyesini yeterli derecede yükseltmek mümkün olmaz. Ablasyon başarısında hastalarda daha düşük düzeyde TSH yükseklikleri yeterli olup, uzun süre hipotiroid dönemde kalınması gerekli olmayabilir.

## INTRODUCTION

Thyroid cancer is the most common endocrine tumor, most of which are papillary thyroid carcinomas. Multidisciplinary treatment of differentiated thyroid cancer (DTC) patients consists of total thyroidectomy followed by radioactive iodine remnant ablation (RRA) and thyroid stimulating hormone (TSH) suppression treatment. RRA is a successful form of treatment that aims to destroy the remaining residual tissue and/or metastatic tissue after surgical treatment in patients with DTC (1). Elevated levels of serum thyroglobulin (Tg) (>2 ng/mL) is a specific indicator with high sensitivity, which indicates presence of residual thyroid tissue, metastatic focus or recurrence (2). Obtaining elevated levels of TSH (thyroid stimulating hormone) (≥30 mIU/L) is recommended for successful ablation (3). High serum TSH concentration enhances I-131 uptake by cancer cells. However, it is not known whether higher TSH levels produce a better rate of remnant ablation or cancer cure. In this retrospective study, our aim was to investigate whether TSH levels during ablation influenced the success of RRA. The secondary aim was to investigate the effect of Tg level at the time of ablation and other clinic and demographic patient related data on ablation success.

## MATERIALS AND METHODS

Patients who were diagnosed with DTC, treated with bilateral total/near total thyroidectomy and who were referred for RRA were included in this retrospective study. Exclusion criteria were patients receiving I-131 treatment in another hospital, patients who were not imaged with whole body scan (WBS) within 8-12th months after ablation, and patients with positive Tg antibodies (TgAb). Activity ranging between 100 to 250 mCi (mean 114±22 mCi) of I-131 were administered orally. The standard therapeutic dose was applied (for ablation therapy: 100 mCi, for lymph node metastasis: 150 mCi, for lung metastasis: 200 mCi, for lung metastasis reablation: 250 mCi). RRA was given to patients who had a TSH level under 30 despite sufficient levothyroxine (LT4) thyroid hormone withdrawal (THW) time (minimum 4 weeks), due to suspicion of metastatic disease. Patients were divided into 2 groups as <30 mIU/L and ≥30 mIU/L according to serum TSH level. Serum TSH, serum Tg and serum TgAb levels were recorded before RRA in all patients after adequate THW for 4-5 weeks. We also recommended a low-iodine diet 10 day before RRA for all patients. The initial clinical follow-up evaluation was performed at the 2nd and 6th months after RRA in all patients. Clinical follow-up included; physical examination, neck ultrasound, and serum Tg, TgAb, TSH, freeT4 measurements. Diagnostic Whole Body Scan (DxWBS) with approximately 185 MBq of I-131, neck ultrasound and chest X-ray, or if required neck and/or chest computed tomography examinations were performed, and serum Tg, TgAb and TSH levels were measured 8-12 months after RRA. Diagnostic WBS was performed 24 and 48 hours after administration of diagnostic dose I-131. TSH-stimulated serum Tg level measurements were obtained at the time of DxWBS performed 8-12 months after ablation in all patients. For stimulated TSH level, the LT4 preparation was stopped 4 weeks before I-131 administration, or recombinant TSH was administered (0.9 mg) by intramuscular injections on two successive days with the I-131 being given on the third day during DxWBS. Scintigraphic images were obtained with the use of a single-headed gamma camera (Toshiba GCA-7100A) that was equipped with a “high-energy parallel hole” collimator and interfaced to a dedicated computer. For image acquisition, a peak energy setting at 364 keV with a 20% window was used. The scan speed was 7 cm/min for all WBS. WBS with anterior and posterior views was acquired, and local static images were obtained. Patients with undetectable thyroid-stimulating hormone-stimulated serum Tg concentrations, normal physical examination, negative results on WBS, and no evidence of neck lymph node metastases on ultrasound were defined as disease-free. The correlation between TSH level at the time of ablation and the success of ablation was evaluated.

**Statistical Analysis**

SPSS 14.0 software was used for statistical analysis. Descriptive quantitative data are expressed as mean values and standard deviation, and qualitative data are expressed as percentages. Correlations between serum TSH and serum Tg levels were examined by the Spearman’s rank correlation test. It was assumed that the observed differences were statistically significant at the p≤0.05 levels. Two-independent samples t-test was used to assess the relationship between success of ablation and levels of serum Tg and serum TSH. We also evaluated the relationship between gender, type of tumor, the number of lesions, age, tumor size, lymph node metastasis at the time of diagnosis and success of ablation.

## RESULTS

Two hundred sixty one consecutive patients were included in the present study. There was 222 (85.1%) female and 39 (14.9%) male patients with a mean age of 45.96±12 years (range; 16-80 years). Hundred and twenty three patients (47.1%) were under the age of 45 and 138 patients (52.9%) were over 45. Thyroid carcinomas were classified as papillary in 205 (78.5%) patients, as follicular in 34 (13%), as thyroid tumors of uncertain malignant potential 15 (5.7%), as poorly differentiated in 4 (1.5%), as aggressive histology (tall cell and insular variant) in 2 (0.8%), and as anaplastic cancer in 1 (0.4%). Mean serum TSH level was 19.47±6 mIU/L in 34 patients with serum TSH level <30 mIU/L, and mean serum TSH level was 73.65±27 mIU/L in 227 patients with serum TSH level ≥30 mIU/L at the time of RRA. In 20.6% of patients with serum TSH level <30 mIU/L, serum Tg level was <2 ng/mL and in 79.4%, serum Tg level was ≥2 ng/mL at the time of RRA. However, in 37.4% of patients with ≥30 mIU/L serum TSH level, serum Tg level was <2 ng/mL and in 62.6% serum Tg level was ≥2 ng/mL (p=0.054) (Table 1). Mean serum Tg level was 43.1 ng/mL (range: 0.10-914 ng/mL) in 34 patients with serum TSH level <30 mIU/L, mean serum Tg level was 19.69 ng/mL (range: 0.08-458 ng/mL) in 227 patients with serum TSH level ≥30 mIU/L (p=0.003). Postoperative stimulated serum Tg levels at the time of ablation therapy were ≤2 ng/mL in 90 patients (34.5%), 2-10 ng/mL in 81 patients (31%) and ≥10 ng/mL in 90 patients (34.5%). Mean stimulated serum Tg level was 7.15 ng/mL (range: 0.10-1000 ng/mL) and mean stimulated-TSH level was 86.11 mIU/mL (range: 12.4-226.5 mIU/mL) at the time of DxWBS. There was a negative correlation between serum TSH level and Tg levels (p=0.007, r=-0.167). Patients with radioactive iodine accumulation outside the thyroid bed (the cervical area or in other areas of the body) or in the thyroid bed region, and with high serum TSH levels (>10 ng/mL or 2-10 ng/mL) were considered as unsuccessful ablation at the time of DxWBS. If there was no significant pathologic radioactive iodine accumulation or minimal local accumulation in the thyroid bed region and if the serum TSH level was low (<2 ng/mL), this was regarded as successful ablation at the time of DxWBS. When all patients were considered, ablation was not successful in 12 patients after the first RRA. Findings of those patients are presented in Table 2. Serum TSH levels were <30 mIU/L in 34 patients (13%) at the time of RRA. Ablation was unsuccessful in only one patient with serum TSH level <30 mIU/L. This patient had lung metastasis, and the serum Tg level was 914 ng/mL at the time of RRA. Reablation was also unsuccessful in the same patient although the serum TSH level was >100 mIU/L at the time of treatment. Ablation was unsuccessful in 5.1% of patients with serum TSH level ≥30 mIU/L. The effect of serum TSH level was not significant on ablation success (p=0.472). There was no significant difference in terms of mean serum TSH levels in patients with and without successful ablation (p=0.472). However, a significant difference was determined in mean serum Tg values (p=0.001) (Table 3). One patient had a serum TSH level <30 mIU/L at the time of both RRA administration and DxWBS obtained 10 months later. No residual tissue or metastatic foci was detected at the latest DxWBS performed. Serum Tg level was <0.20 ng/mL both at the time of low dose scanning scintigraphy and during follow-ups. No abnormal finding was detected clinically and radiologically. Gender, type of tumor, the number of lesions (multifocal or single) were not found to be significantly associated with RRA outcome (p=0.086, p=0.848, p=0.524, respectively). Tumor size and lymph node metastasis at the time of diagnosis were found to be significantly associated with RRA (p=0.002, p=0.0001, respectively). Also, age at the time of diagnosis was significantly associated with RRA (p=0.0001). While ablation was successful in all patients younger than 45 years, ablation was unsuccessful in 8.7% of patients older than 45 years.

## DISCUSSION

RRA is a safe and effective method which has been used for a long time in the treatment of DTC patients with total thyroidectomy. There are no controlled studies that assess the adequate level of endogenous TSH for optimal ablation therapy. However, when treating a patient with radioactive iodine, it is important to stimulate iodine uptake by elevating serum TSH levels prior to radioactive iodine administration. The recommended TSH level is ≥30 mIU/L (1). Because, the clearance of radioactive iodine is approximately 50% greater in euthyroid patients than in hypothyroid patients (4), a high serum TSH concentration enhances I-131 uptake by cancer cells. TSH stimulates the production and release of thyroid hormones as well as stimulating Tg production (5). Prolonged hypothyroidism is undesirable both due to hypothyroidism symptoms and the risk of stimulating tumor growth. Tg is a significant tumor marker for DTC patients. Prior to I-131 therapy, LT4 replacement must be discontinued for approximately 4-5 weeks to achieve an adequate TSH level, or TSH can be stimulated by recombinant human TSH (rhTSH) without discontinuing thyroid hormone therapy. A higher level of TSH can be obtained with rhTSH application as compared to THW protocol (6). Nevertheless, rhTSH is not yet recommended as the standard therapeutic for the purpose of RRA in metastatic thyroid cancer. TSH stimulated Tg measurement is compulsory to achieve sufficient clinical sensitivity for the detection of persistent and/or recurrent disease for current clinical guidelines. There are studies in the literature analyzing both the required period for ensuring adequate TSH levels and the optimal TSH level in order to reach a sufficient Tg level. Sánchez et al. (7) showed that TSH increases to >30 mIU/L in 90% of patients 3 weeks after discontinuing LT4 suppressive therapy. Luna et al. (8) also claimed that discontinuation of thyroxine treatment for four weeks was not required. According to them, a fourteen day period was adequate in most patients, and 21 days were sufficient in almost all. Similarly, Serhal et al. (5) stated that discontinuing thyroid hormone preparations for 2-3 weeks provided adequate iodine uptake. Goldman et al. (9) reported that in patients using LT3, withdrawal for 2 weeks produced the same effect as 4 week drug interruption even in metastatic patients. Valle et al. (10) determined that TSH cutoff of ≥30 mIU/L was inadequate to detect patients with thyroid-stimulating hormone-stimulated serum Tg ≥2 ng/mL, while TSH >80-100 mIU/L was a better cut off. However, there is still no consensus on the TSH value to obtain the highest Tg level. Low serum Tg level at the time of ablation has a negative predictive value for the absence of residual disease, and the risk of persistent disease increases with stimulated Tg levels (11). Postoperative stimulated Tg level is primarily related to surgeon success, and the presence of refractory disease or normal thyroid remnant. Absence of residual thyroid tissue is extremely rare even after successful total thyroidectomy applied by experienced surgeons. In patients with total thyroidectomy followed by I-131 ablation for DTC, the baseline stimulated-Tg level is a good predictor of successful ablation (12). In the literature, some studies have reported that the serum Tg/serum TSH ratio was an important predictor of ablation success that correlated well with patient outcomes. Moreover, they suggested that this rate and similar laboratory parameters might be considered while determining risk stratifications of DTC patients (13,14). Although a high TSH level (≥30 mIU /L) is recommended for ablation success in all textbooks, to the best of our knowledge there is only one study that assesses the correlation between ablation success and low TSH level (<30 mIU/L) in the literature. Vrachimis et al. (15), reported in their study on 1.873 patients without distant metastases that endogenous TSH levels at the time of I-131 ablation were not correlated with ablation success rates, recurrence free survival or DTC related mortality. TSH level was <30 mIU/L in 275 of patients in that study. It is known that, TSH elevation is slow or minimal in the presence of large residual tissue after total thyroidectomy, or in the presence of metastatic disease. If Tg level is low (<2 ng/mL) then TSH levels are known to rise easily. A high Tg level indicates presence of large residual tissue or metastasis. Therefore, the adequate TSH levels may not be reached especially in metastatic patients and in patients with large residual tissue even if the T4 preparation is discontinued for longer periods. Besides, if patients have malignant struma ovarii or hypopituitarism, TSH level will not elevate (16). Sawicka-Gutaj et al. (17) reported that the preablative TSH level in DTC patients with pyramidal lobe was statistically lower than the control group. However, TSH level was not different between DTC patients with and without pyramidal lobe 1 year after RRA. Moreover, although high TSH levels with recombinant TSH can be obtained quickly there are doubts about sufficient iodine uptake. Due to the retrospective nature of our study, weekly TSH levels were unfortunately not measured. In this study, patients for whom thyroid hormone preparations were discontinued for 4-5 weeks and were treated even though having TSH levels <30 mIU/L at the day of RRA were evaluated. In our study, the ablation was unsuccessful in one out of 34 patients with TSH level <30 mlU/L. But serum Tg level was 914 ng/mL despite the low TSH level. The treatment was unsuccessful although TSH level was >100 mIU/L during reablation in the same patient. Ablation was unsuccessful in 5.1% of patients with TSH ≥30 mIU/L. TSH level did not show a significant effect on ablation success. Presence of I-131 uptake by tumor, younger age, well differentiated histopathologic subtype, and presence of metastases are predictive factors for tumor response to radioiodine treatment (1). Serum TSH level gradually decreases with age (18). In our study, all patients who had unsuccessful ablation were above the age of 45. Age may be one of the reasons for not obtaining a desired TSH level. Interestingly, Montesano et al. (19) have determined that higher TSH levels can be achieved after rhTSH application in patients with advanced age. Besides, when considered together with Tg levels, the reason for the low TSH level may be related to the presence of residual tissue and/or metastatic disease. In such situations, extension of hypothyroid period does not contribute in terms of iodine uptake. In our study, the ablation was not successful in almost all patients who had metastatic disease. In one of these patients, ablation was unsuccessful due to large residual tissue. The level of cell differentiation is as much important as the volume of residual tissue in iodine efficiency. Additionally, there is no significant evidence that rapid tumor growth is stimulated by a brief rise in TSH concentration (4). Individual iodine supply is also important. When all these factors are considered in combination, it may be concluded that the TSH level may not necessarily point out RRA activity.

## CONCLUSION

In conclusion, we think that a high serum TSH level is not enough for the success of ablation by itself. The success of ablation at lower levels of TSH elevations may be sufficient for patients and long-term hypothyroidism may not be required. Instead of assessing TSH or Tg level alone before RRA; age, presence of metastasis, extent of residual thyroid mass should also be considered. RRA may still be performed even if TSH remains low despite a sufficient period of THW.

## Ethics

Ethical Approval: This study was retrospective. All procedures performed in studies involving human participants were in accordance with the ethical standards of the institutional research committee and with the 1964 Helsinki Declaration and its later amendments or comparable ethical standards. This article does not contain any studies with human participants performed by any of the authors.

Peer-review: Externally peer-reviewed.

Financial Disclosure: The authors declared that this study has received no financial support.

## Figures and Tables

**Table 1:**
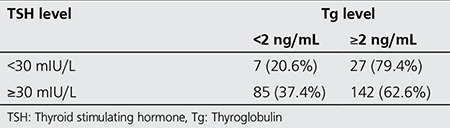
Comparison of serum thyroid stimulating hormone and thyroglobulin level

**Table 2:**
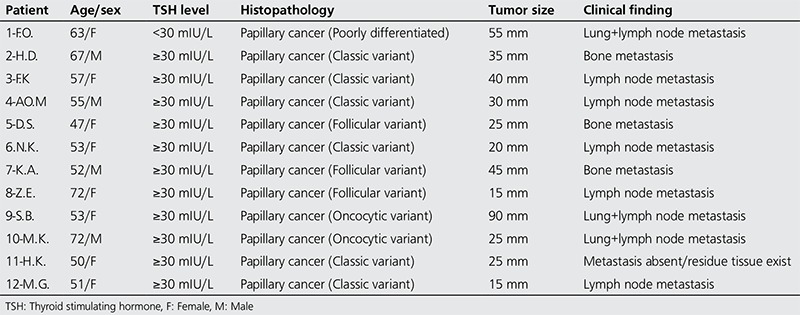
Clinicopathologic findings of 12 patients in whom ablation was unsuccessful after the first radioiodine remnant ablation

**Table 3:**
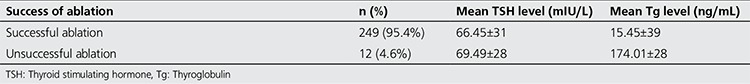
Comparison of serum thyroid stimulating hormone and thyroglobulin levels at the time of ablation according to ablation success
